# A cross-sectional study of the association between ventilation of gas stoves and chronic respiratory illness in U.S. children enrolled in NHANESIII

**DOI:** 10.1186/1476-069X-13-71

**Published:** 2014-09-02

**Authors:** Molly L Kile, Eric S Coker, Ellen Smit, Daniel Sudakin, John Molitor, Anna K Harding

**Affiliations:** College of Public Health and Human Sciences, Oregon State University, 15 Milam, Corvallis, OR 97331 USA; Environmental and Molecular Toxicology, College of Agricultural Sciences, Oregon State University, Corvallis, OR 97331 USA

**Keywords:** Asthma, Wheeze, Bronchitis, Gas stoves, Ventilation, Spirometry, NHANES, Children

## Abstract

**Background:**

Gas stoves emit pollutants that are respiratory irritants. U.S. children under age 6 who live in homes where gas stoves are used for cooking or heating have an increased risk of asthma, wheeze and reduced lung function. Yet few studies have examined whether using ventilation when operating gas stoves is associated with a decrease in the prevalence of respiratory illnesses in this population.

**Methods:**

The Third National Health and Nutrition Examination Survey was used to identify U.S. children aged 2–16 years with information on respiratory outcomes (asthma, wheeze, and bronchitis) who lived in homes where gas stoves were used in the previous 12 months and whose parents provided information on ventilation. Logistic regression models evaluated the association between prevalent respiratory outcomes and ventilation in homes that used gas stoves for cooking and/or heating. Linear regression models assessed the association between spirometry measurements and ventilation use in children aged 8–16 years.

**Results:**

The adjusted odds of asthma (Odds Ratio [OR] = 0.64; 95% confidence intervals [CI]: 0.43, 0.97), wheeze (OR = 0.60, 95% CI: 0.42, 0.86), and bronchitis (OR = 0.60, 95% CI: 0.37, 0.95) were lower among children whose parents reported using ventilation compared to children whose parents reported not using ventilation when operating gas stoves. One-second forced expiratory volume (FEV_1_) and FEV_1_/FVC ratio was also higher in girls who lived in households that used gas stoves with ventilation compared to households that used gas stoves without ventilation.

**Conclusions:**

In homes that used gas stoves, children whose parents reported using ventilation when operating their stove had higher lung function and lower odds of asthma, wheeze, and bronchitis compared to homes that never used ventilation or did not have ventilation available after adjusting for other risk factors. Additional research on the efficacy of ventilation as an intervention for ameliorating respiratory symptoms in children with asthma is warranted.

**Electronic supplementary material:**

The online version of this article (doi:10.1186/1476-069X-13-71) contains supplementary material, which is available to authorized users.

## Background

Asthma is the most common chronic childhood disease and is characterized by recurrent airway obstruction, bronchial hyper-responsiveness, and airway inflammation [1]. It is also the leading cause of childhood hospitalizations and school absenteeism [2]. There is considerable evidence that air pollution - specifically coarse and fine particulate matter, ozone, sulfur dioxides, and nitrogen oxides - is associated with increased rates of asthma, asthma morbidity, respiratory illness and diminished lung function in children [3–8].

In the indoor environment, gas stoves are a common source of air pollution, including combustion-related particulate matter and nitrogen oxides [9–16]. While gas stoves are primarily used for cooking, approximately 7.7 million U.S. households (9.3%) reported using their gas stove or oven for heat at least once during the previous year [17]. There is considerable evidence from epidemiological studies in developed countries that gas stoves used for cooking and/or heat are associated with an increased risk of asthma and respiratory symptoms in children [9, 18–33]. Although other studies that have examined the relationship between gas stoves or nitrogen dioxide levels in homes do not observe significant associations with asthma symptoms in childhood [34–36].

Housing characteristics have been shown to influence indoor air pollution levels. For instance, indoor air concentrations of nitrogen dioxide can be higher than ambient levels if there are unvented combustion appliances in the home, such as gas stoves [37]. Ventilation has also been shown to reduce the concentration of other indoor air pollutants such as formaldehyde and volatile organic compounds [38]. There are many different types of household ventilation systems, some of which are automatic, and some of which require point-of-use operation such as kitchen stove vent hoods. While several studies have examined the role of ventilation on indoor air pollutants and indoor air pollution on children’s chronic respiratory illnesses, little is known about the role of behavior related to point-of-use ventilation and how this behavior might influence children’s respiratory health [39–41]. Subsequently, we theorized that if gas stoves in homes and their emissions are related to asthma and its symptoms in children, then using ventilation when operating gas stoves should reduce indoor air pollution levels and benefit children’s respiratory health outcomes. Specifically, we hypothesized that using ventilation when operating gas stoves should be associated with a lower prevalence of chronic respiratory illnesses in children.

## Methods

### Study population

The Third National Health and Nutrition Examination Survey (NHANES III) is a nationally representative cross-sectional survey of the civilian non-institutionalized U.S. population conducted by the National Center for Health Statistics from 1988–1994. Participants were administered standardized interviews in their homes and underwent physical examinations and laboratory testing in mobile examination centers [42]. NHANES III includes data on children’s respiratory health, spirometry data and residential characteristics, which provide a unique opportunity to assess the relationship between parental habits when using gas stoves and respiratory illnesses in U.S. children.

To focus on the association between respiratory illnesses in children and parental use of ventilation in homes that had gas stoves in their kitchens, the current analysis was restricted to children aged 2–16 years (n = 12,570) whose parents: *i)* reported that a gas stove was used in the past twelve months in their child’s primary residence (yes); *ii)* provided information on the presence of ventilation near the gas stove (yes/no) and their use of ventilation (never, rarely, sometimes, or always); *iii)* provided information on their child’s respiratory health (doctor-diagnosed asthma [yes/no], doctor-diagnosed bronchitis [yes/no], and chest wheeze [yes/no]); and *iv)* reported their child’s body mass index, parental history of asthma or hay fever (yes/no), presence of a pet in the household (yes/no), and history of smoking cigarettes indoors (yes/no). Twelve respondents did not answer the question about ventilation. Fewer participants consented to the examination portion of the survey where measurements were taken to compute body mass index. This resulted in data on 7,378, 7,380, and 7,378 children who resided in a home that had a gas stove in the kitchen and who provided information on asthma, wheeze, and chronic bronchitis respectively. Additionally, spirometry measurements were measured only in a subset of children ≥ 8 years old (N = 2,400). Details on deriving the sample size are provided in Additional file 1: Figure S1. Missing data was assumed to be completely at random.

NHANES III was approved by the National Center for Health Statistics Institutional Review Board. Participants who were 12 to 17 years old and their parents provided informed consent; participants who were 7 to 11 years old provided assent and their parents provided consent; and, parents provided informed consent for those <7 years old.

### Behaviors when using gas stoves and ventilation characterization

Parents were asked, “Is there a gas stove or oven used to cook in this house (yes/no).” Only parents who answered “yes” were asked the follow up question about ventilation. Due to this skip pattern design in the NHANES III questionnaire, the analytical sample was restricted to children whose parents answered “yes” to the gas stove question. Children were classified as living in households that used gas stoves for heat (yes/no) based on their parent’s response to the question, “Was this gas stove or oven used to heat the house over the past 12 months (yes/no)”.

Ventilation was characterized based on parent’s response to, “Is there an exhaust fan near this stove that sends fumes outside the home (yes/no)” and, “How often is this exhaust fan used (never, rarely, sometimes, or always).” We classified children as living in a household that did not use ventilation if parents stated that there was no exhaust fan or that they never used the exhaust fan. We classified children as living in a household that used ventilation if parents reported that they rarely, sometimes or always used the exhaust fan.

### Respiratory health outcomes

For children aged 2–16 years of age, dichotomous respiratory health outcomes were available including parent-reported: *i)* doctor-diagnosed asthma, *ii)* chest wheeze or whistling in past 12 months, and *iii)* doctor-diagnosed chronic bronchitis.

Lung function tests were performed at the mobile examination centers on children between 8–16 years of age following spirometry protocols issued by the American Thoracic Society [43].

### Sociodemographic factors and covariates

Selected characteristics were assessed for their relationship to respiratory outcomes and parental behaviors regarding gas stoves. These included age group, sex, race-ethnicity, parental education, parental history of asthma or hay fever, body mass index percentile for age cut-offs following the U.S. Centers for Disease and Prevention recommended guidelines [44], poverty income ratio, household income < $20,000, cigarette smoking indoors, heating with a gas stove, the presence of pets in the household (only cats, dogs and birds), type of residence (rural versus urban) and US census region.

### Statistical approach

To account for the complex sampling design, data were analyzed using appropriate NHANES sample weights using the “svy” command in Stata version 12.1 (StataCorp, College Station, TX). The weighted proportions of participants with respiratory health outcomes and 95% confidence intervals were calculated for children residing in four different settings in homes: (1) where parents reported using ventilation when operating gas stoves for cooking or heating; (2) where parents reported not using ventilation when operating a gas stove for cooking or heating; (3) where parents reported using ventilation when operating gas stoves for cooking only; and, (4) where parents reported not using ventilation when operating gas stoves for cooking only. Chi-squared tests assessed the association between prevalent respiratory health outcomes and ventilation use. Covariates were included in the models if they were associated with a respiratory health outcome at α < 0.20. Additionally, household income below $20,000, which had the least amount of missing data, was included in each model because prior research has demonstrated a strong association between income and reported ventilation use.

Multivariate linear regression models assessed the association between percent of predicted spirometry measurements (one-second forced expiratory volume [FEV_1_], forced vital capacity [FVC], and FEV_1_/FVC ratio) and gas stoves in all children aged 8–16 years. These models were also stratified by sex. Reference population spirometry values were calculated using NHANES III race and sex specific estimating equations that accounted for age and height for FEV_1_ and FVC, as derived by Hankinson et al. [45] and Collen et al. [46]. Percent-predicted values were calculated by taking the ratio of observed spirometry measurements over predicted values and multiplying by 100%. Model covariates accounted for environmental and host factors such as indoor cigarette smoking, the presence of pets in the home, household income < $20,000, using a gas stove for heating purposes, and asthma status.

## Results

Population characteristics and prevalence rates of respiratory illnesses for children residing in homes that used a gas stove are presented in Table 1. Overall, the unadjusted prevalence of wheeze (14.2% vs. 19.3%, p-value = 0.01, N = 7,380) and bronchitis (3.2% vs. 5.0%, p-value = 0.02, N = 7,378) were lower among children residing in households that reported using ventilation when operating their gas stoves compared to households that did not use ventilation when operating their gas stove. The unadjusted prevalence of asthma (8.1% vs. 11.1%, p-value = 0.11, N = 7,378) was not significantly different between households by ventilation status. The unadjusted prevalence of asthma (8.86% vs. 13.54%, p = 0.04) and wheeze (15.7% vs. 23.26%, p = 0.003), but not bronchitis (3.94% vs. 4.48%, p = 0.62), was lower among children residing in households that reported not using a gas stove for heat compared to households that used a gas stove for heat. In unadjusted models, asthma prevalence was also associated with gender, BMI, parental history of asthma or hay fever, household income < $20,000, and age group. The unadjusted prevalence of chronic bronchitis was associated with age, race-ethnicity, parental history of asthma or hay fever, indoor cigarette smoke, household income < $20,000, and census region. The unadjusted prevalence of wheeze was associated with age, parental history of asthma or hay fever, the presence of a pet with fur or a bird in the home, indoor cigarette smoke, race-ethnicity, household income < $20,000, and BMI. Parental education and urban versus rural residence were not associated with any health outcomes (data not shown).

**Table 1:** **Mean percent prevalence with 95% confidence intervals of asthma, wheeze, and bronchitis among children aged 2–16 living in homes with gas stoves by different stove use habits** ^**1**^

	Ever diagnosed with asthma (N = 7,390)			Wheeze in past 12 months (N = 7,392)			Ever diagnosed with chronic bronchitis (N = 7,390)		
Variable	No. cases	Prevalence (95% CI)	***P-value*** ^***2***^	No. cases	Prevalence (95% CI)	***P-value*** ^***2***^	No. cases	Prevalence (95% CI)	***P-value*** ^***2***^
Total cases	572	9.48 (8.87, 11.30)		1,422	16.58 (14.88, 18.28)		329	4.05 (3.08, 5.03)	
**Vent used w/gas stove**		*n = 7,378*			*n = 7,380*			*n = 7,378*	
Yes	260	8.07 (6.11, 10.59)	*0.11*	643	14.20 (11.79, 17.01)	*0.01*	134	3.17 (2.20, 4.53)	*0.02*
No	310	11.09 (8.42, 14.47)		776	19.30 (16.90, 21.95)		194	5.08 (3.85, 6.67)	
**Gas stove used for heating**		*n = 7,346*			*N = 7,348*			*n = 7,346*	
Yes	117	13.54 (9.46, 19.03)	*0.04*	278	23.26 (18.35, 29.03)	*0.003*	74	4.48 (2.73, 7.25)	*0.62*
No	447	8.86 (7.16, 10.92)		1,133	15.70 (14.05, 17.52)		251	3.94 (3.03, 5.09)	
**Vent and Stove Use**		*n = 7,334*			*n = 7,336*			*n = 7,334*	
Vent not used and stove used for cooking and heating	78	13.63 (8.59, 20.97)	*0.13*	197	25.07 (18.58, 32.91)	*0.003*	60	5.43 (3.51, 8.33)	*0.10*
Vent used and stove used for cooking and heating	39	13.40 (7.41, 23.03)		81	20.14 (13.84, 28.38)		14	2.82 (1.09, 7.08)	
Vent not used and stove only used for cooking	224	10.36 (7.48, 14.18)		*568*	18.13 (15.59, 20.99)		130	4.87 (3.52, 6.72)	
Vent used and stove only used for cooking	221	7.67 (5.63, 10.35)		*562*	13.76 (11.32, 16.63)		120	3.20 (2.19, 4.65)	

^1^Unweighted sample sizes and weighted proportions (prevalence).

^2^P-values obtained from χ2 test.

Multivariate logistic regression models were used to evaluate the odds of chronic respiratory illnesses in children who lived in homes where gas stoves were ventilated and only used for cooking while adjusting for other confounders (Table 2: Model 1). After adjusting for confounders, children who lived in homes where parents reported that they used ventilation were less likely to be diagnosed with asthma (aOR = 0.64, 95% CI: 0.43, 0.97), diagnosed with chronic bronchitis (aOR = 0.60, 95% CI: 0.37, 0.95), or report wheeze (aOR = 0.60, 95% CI: 0.42, 0.86). When parental habits regarding using a gas stove for heating were included as an additional covariate (Table 3: Model 2), only wheeze (aOR = 0.62, 95% CI: 0.44-0.89) and chronic bronchitis (aOR = 0.61, 95% CI: 0.38-0.98) remained significantly associated with vented gas stoves after adjusting for other confounders (p = 0.01 and p = 0.04, respectively). In homes where parents reported using gas stoves only for cooking and not heating, children were significantly less likely to have a diagnosis of asthma (aOR = 0.56, 95% CI: 0.34-0.94) and wheeze (aOR = 0.57, 95% CI: 0.38-0.85), compared to children in homes that used a gas stove for cooking and heating after adjusting for other confounders. The odds of chronic bronchitis, however, was not significantly different for households that used a gas stove only for cooking compared to households that used a gas stove for cooking and heating (aOR = 1.12, 95% CI: 0.66-1.92) after adjusting for other confounders.

**Table 2:** **Adjusted Odds ratios and 95% confidence intervals for the association between respiratory illnesses in children aged 2–16 years who live in households that use gas stove with ventilation compared to households that use gas stoves without ventilation (Model 1)**

	Ever diagnosed with asthma ^a^(N = 5,745)		Wheeze in past 12 months ^b^(N = 5,744)		Ever diagnosed with bronchitis ^c^(N = 7,255)	
Ventilation of gas stove	No. cases	OR (95% CI)	No. cases	OR (95% CI)	No. cases	OR (95% CI)
No	*269*	1 Ref.	*561*	1 Ref.	*188*	1 Ref.
Yes	*224*	0.64 (0.43, 0.97)*	*458*	0.60 (0.42, 0.86)*	*128*	0.60 (0.37, 0.95)*

*P-value <0.05.

^a^Adjusted for age group, sex, parental history of asthma or hay fever, and furry or feathery pets in the house, household income < $20,000, and BMI percentiles for age.

^b^Adjusted for age group, parental history of asthma or hay fever, furry or feathery pets in the house, indoor tobacco smoke, race-ethnicity, household income < $20,000, and BMI percentile for age.

^c^Adjusted for age group, parental history of asthma or hay fever, indoor tobacco smoke, race-ethnicity, household income < $20,000, and census region.

**Table 3:** **Adjusted Odds ratios and 95% confidence intervals for the association between respiratory illnesses in children aged 2–16 years and gas stove use habits (Model 2)**

	Ever diagnosed with asthma ^a^(N = 5,646)		Wheeze in past 12 months ^b^(N = 5,647)		Ever diagnosed with bronchitis ^c^(N = 7,114)	
	***No. cases***	OR (95% CI)	***No. cases***	OR (95% CI)	***No. cases***	OR (95% CI)
**Gas stove used with ventilation**						
No	*263*	1 Ref.	*555*	1 Ref.	*184*	1 Ref.
Yes	*224*	0.68 (0.45, 1.04)	*458*	0.62 (0.44, 0.89)*	*128*	0.61 (0.38, 0.98)*
**Gas stove used for heating**						
Yes	*382*	1 Ref.	*795*	1 Ref.	*239*	1 Ref.
No	*105*	0.56 (0.34, 0.94)*	*218*	0.57 (0.38, 0.85)*	*73*	1.12 (0.66, 1.92)

*P-value <0.05.

^a^Adjusted for gas stove used for heating, age group, sex, parental history of asthma or hay fever, and pets in the house, household income < $20,000, and BMI.

^b^Adjusted for gas stove used for heating, age group, parental history of asthma or hay fever, pets in the house, indoor tobacco smoke, race-ethnicity, household income < $20,000, and BMI.

^c^Adjusted for gas stove used for heating, age group, parental history of asthma or hay fever, indoor tobacco smoke, race-ethnicity, household income < $20,000, and census region.

Table 4 examined the potential for joint effects of ventilation practices and using the gas stove for heating on respiratory illness in children. Compared to children living in homes where parents reported not using ventilation and who also used the gas stove for heat, using ventilation lowered the odds of asthma in children by 14%; not using the stove for heat lowered the odds by 38%; and using ventilation and not using the stove for heat lowered the odds by 59%. Similar results were found for wheezing. However, no significant association was observed for the joint effect of ventilation and using the gas stove heat on the odds of chronic bronchitis.

**Table 4:** **Adjusted Odds ratios and 95% confidence intervals for respiratory illnesses in children aged 2–16 years and the joint association between ventilation (yes/no) and gas stove use habits (cooking only/cooking and heating)**

	Asthma ^a^(N = 5,646)		Wheeze ^b^(N = 5,647)		Bronchitis ^c^(N = 7,114)	
	***No. cases***	aOR (95% CI)	***No. cases***	aOR (95% CI)	***No. cases***	aOR (95% CI)
Vent not used and stove used for cooking & heating	*69*	1 Ref.	*156*	1 Ref.	*59*	1 Ref.
Vent used and stove used for cooking & heating	*36*	0.86 (0.34, 2.17)	*62*	0.62 (0.31, 1.20)	*14*	0.49 (0.21, 1.12)
Vent not used and stove used only for cooking	*194*	0.62 (0.32, 1.23)	*399*	0.57 (0.35, 0.92)*	*125*	1.05 (0.61, 1.81)
Vent used and stove used only for cooking	*188*	0.41 (0.23, 0.74)*	*396*	0.35 (0.21, 0.60)*	*114*	0.65 (0.36, 1.19)

*P-value <0.05.

^a^Adjusted for age group, sex, parental history of asthma or hay fever, household income < $20,000, pets in the house, and BMI.

^b^Adjusted for age group, parental history of asthma or hay fever, pets in the house, indoor tobacco smoke, race-ethnicity, household income < $20,000, and BMI.

^c^Adjusted for age group, parental history of asthma or hay fever, indoor tobacco smoke, race-ethnicity, household income < $20,000, and census region.

The relationship between lung function and behavioral factors related to gas stoves are presented in Table 5. The FEV_1_ measurements ranged between 468 mL to 5683 mL with a weighted mean and standard deviation of 2658 mL and 882 mL. The FVC measurements ranged between 864 to 6846 mL with a weighted mean and standard deviation of 3069 mL and 1036 mL. For the FEV_1_/FVC ratio, we observed a range between 31.6% and 100% with a weighted mean and standard error of 86.9% and 0.2%. Among children aged 8–16 years who provided spirometry measurements, unadjusted mean FEV_1_ and FVC were higher in children who lived in homes where parents used an exhaust vent compared to children who lived in homes where there was no exhaust vent or parents reported not using the exhaust vent when operating their gas stoves (Table 5). Table 6 compares the percent of predicted (or normalized) differences in spirometry measurements among children aged 8–16 years in households that operated gas stoves with ventilation compared to households that operated gas stoves without ventilation. In fully adjusted models, the overall percent-predicted FEV_1_ (p = 0.08), FVC ( p = 0.20) and FEV_1_/FVC (p = 0.11) were modestly higher in children living in homes with vented gas stoves compared to homes without ventilation of gas stoves, although these did not reach statistical significance (Table 6). Although after stratifying by sex, we observed that the percent-predicted FEV_1_ was almost 3% higher in girls (p = 0.02) that lived in homes where parents reported using ventilation compared to homes where ventilation was not used. There was no significant association between venting of gas stoves with FVC in girls (p = 0.13). The percent-predicted FEV_1_/FVC ratio was 1.6% (95%CI: 0.16, 3.0, p-value = 0.03) higher among girls living in homes that reported vent usage compared to girls in homes that reported not using ventilation with gas stoves (Table 6). No associations between spirometry measurements and ventilation were observed in boys. In addition, no association between spirometry and heating with a gas stove were observed overall or in the sex-stratified analysis.

**Table 5:** **Univariate association between behaviors related to gas stove use and spirometry measurements for FEV**_**1**_ **(mL), FVC (mL) and FEV**_**1**_ **/FVC Ratio in children aged 8–16 years**

	***N***	Mean FEV _1_(95% CI)	Mean FVC (95% CI)	FEV _1_/FVC (95% CI)
All	*2,472*	2658 (2586, 2730)	3069 (2977, 3161)	86.9% (86.5, 87.3)
Vented Gas Stove				
Yes	*1,147*	2742 (2645, 2841)*	3147^†^ (3027, 3267)	87.4% (86.8, 88.0)
No	*1,325*	2562 (2457, 2668)	2981 (2850, 3113)	86.4% (85.8, 87.1)
Gas Stove Used for Heating				
Yes	*441*	2569 (2385, 2755)	2963 (2751, 3175)	86.9% (85.9, 87.9)
No	*2,017*	2670 (2595, 2744)	3084 (2989, 3179)	86.9% (86.5, 87.3)

*p-value ≤0.05.

^†^0.05 < p-value ≤ 0.1.

**Table 6:** Differences in percent of predicted spirometry (observed/predicted*100%) indicators among children aged 8–16 years in households that operated gas stoves with ventilation compared to households that operated gas stoves without ventilation that is stratified by gender (females N = 1,192; males N = 1,186)

		FEV _1_(Crude)	FEV _1_(Adjusted) ^a^	FVC (Crude)	FVC (Adjusted) ^a^	FEV _1_/FVC (Crude)	FEV _1_/FVC (Adjusted) ^a^
		Difference ^b^(95% CI)	Difference ^b^(95% CI)	Difference ^b^(95% CI)	Difference ^b^(95% CI)	Difference ^b^(95% CI)	Difference ^b^(95% CI)
	*N*	*2,378*	*2,335*	*2,3378*	*2,335*	*2,378*	*2,335*
All	*1113*	2.75 (0.29, 5.21)*	2.33 (-0.29, 4.95)	2.08 (-0.66, 4.82)	1.75 (-0.95, 4.44)	1.14 (-.03, 2.31)	0.97 (-0.24, 2.17)
Female	*570*	2.86 (0.71, 5.01)*	2.93 (0.57, 5.30)*	1.6 (-0.4, 3.6)	1.76 (-0.51, 4.02)	1.45 (0.05, 2.85)*	1.58 (0.16, 3.00)*
Male	*543*	2.62 (-1.36, 6.61)	1.74 (-1.74, 5.24)	2.24 (-2.47, 6.96)	1.59 (-2.14, 5.32)	0.87 (-0.56, 2.30)	0.43 (-0.94, 1.81)

^a^Adjusted for environmental tobacco smoke, using a gas stove for heating, furry or feathery pets in the home, asthma status and household income < $20,000.

^b^NHANESIII reference spirometry measurements derived from Hankinson et al. [45].

*P-value <0.05.

## Discussion

The results show that among children who live in households with a gas stove kitchen appliance, the prevalence of respiratory illness was lowest in children when ventilation was used when operating the gas stove and when the gas stove was not used for heat. Our finding support previous analysis of NHANES III by Lanphear et al. [28], which found that using a gas stove for heating increased the likelihood of asthma in children. Our analysis suggests that ventilation is likely an effect modifier of this association. Furthermore, we observed better lung function in children living in households where ventilation was used when operating the gas stove than in households that did not have ventilation or where no ventilation was used. This association with lung function was only significant in girls and it is unclear whether this stems from a greater sensitivity to gas stove emissions or differential behaviors that would result in more frequent exposure to gas stoves. Children’s lung function, however, was not associated with parental report of using the gas stove for heat.

While indoor air pollution measurements are not available in NHANESIII, there is considerable evidence that gas stoves emit pollutants that adversely impact respiratory health and lend biological plausibility to our findings. Gas cooking and heating are a major source of nitrogen dioxide in the indoor environment [34–36]. In animal models, dose-dependent effects of nitrogen dioxide include activation of nuclear factors (NF-kB) within airway epithelial cells, resulting in neutrophilic inflammation and increased release of inflammatory cytokines [47]. Other mechanistic studies have consistently described that nitrogen dioxide has adjuvant properties in the development of allergic asthma by promoting eosinophilia, and the production of antigen-specific IgE and IgG antibodies [48]. In epidemiological studies, short- and long-term exposure to nitrogen dioxide has been inversely associated with FEV1 in pediatric populations [49, 50]. A recent prospective epidemiological study found a higher risk of asthma morbidity among asthmatic children exposed to nitrogen dioxide levels below the US EPA outdoor air standard [51]. Polycyclic aromatic hydrocarbons (PAHs), another pollutant emitted from gas stoves, is also known to augment the allergic response by enhancing the release of inflammatory mediators in the immune system [52, 53]. Polycyclic aromatic hydrocarbons are commonly found in association with fine particulate matter (PM_2.5_), which has been inversely associated with FEV_1_ in preschool children [54]. A recent case–control study in children found strong associations between environmental exposure to PAHs and multiple asthma-related biomarkers including IgE and inflammatory cytokines [55].

Using an exhaust fan can improve indoor air quality and reduce pollutants generated from gas stoves [9–16, 56–58]. Thus, it is plausible that children who live in households that use exhaust fans when operating their gas stoves have better lung function and lower odds of respiratory illnesses. The assessment of the presence or absence of an exhaust fan in homes with gas stoves may be an important environmental factor to consider when taking an exposure history. Physicians, nurses, or health educators could encourage parents to use exhaust fans when operating gas stoves as an additional intervention for improving their children’s respiratory health. Further, physicians, nurses and health educators could discourage the use of a gas stove as a household heating source.

It is important to note that this study has several limitations. While the study is generalizable to all U.S. non institutionalized children ages 2–16 years of age, it is cross-sectional and so we cannot comment on the temporal relationship between households with gas stoves, parental use of ventilation, and respiratory illnesses. NHANES III does not measure indoor air pollution levels which also limits our ability to quantitatively evaluate the relationship between gas stove emissions, ventilation practices, and respiratory outcomes. This analysis did not control for ambient air pollution concentrations because this data is not collected in NHANES and while it is possible to link NHANES data to ambient air pollution this would require access to restricted data that was outside the scope of this study. Nor did this survey collect information on the specific type of ventilation system or its effectiveness. Collecting information on the types of ventilation and its effectiveness by quantitatively measuring indoor air pollution in a nationally representative survey, like NHANES, would be very useful for future studies examining the relationship between gas stoves and respiratory health. Additionally, both the exposures and the outcomes in this study relied upon parental recall which may be a source of bias. It is therefore possible that respondents under-reported smoking behaviors which could explain why indoor smoke exposure was not a risk factor for asthma even though exposure to environmental smoke exposure was a risk factor for bronchitis in this sample. However, the consistency of our results between parental-reported respiratory illnesses in children and quantitative lung function measurements provide additional confidence in the association between ventilation practices and children’s respiratory health. There were also missing observations, particularly for BMI because fewer people consent to the physiological measurement portion of the survey. However, when we analyze the data without BMI using the larger sample size, the statistical significance of the observed associations did not change in any meaningful way for asthma or wheeze (data not shown). Missing data could lead to selection bias but the consistency in the results (with or without BMI) makes this seem unlikely. Finally, the survey only queried respondents about ventilation if they indicated that they had a gas stove making it impossible to evaluate the effect of ventilation on respiratory outcomes in homes that electric stoves. Moreover, we opted to categorize ventilation usage using an extreme dichotomy (no exhaust fan or never use exhaust fan versus rarely, sometimes and always using exhaust fan) rather than four gradations of ventilation use (never, rarely, sometimes and always) because the division between rarely and sometimes is somewhat ambiguous and only 15 people with asthma and 17 people with bronchitis reported “rarely” using their exhaust fan.

## Conclusion

This study observed that using a ventilating exhaust fan when operating a gas stove for cooking or heating was associated with a lower prevalence of asthma and other chronic respiratory symptoms in U.S. children after adjusting for other risk factors. Ensuring that ventilation is installed near gas stoves and that it is used when operating gas stoves is important, as is, only using gas stoves for cooking and not as an auxiliary heat source. The built environment and how people interact with their built environment, such as gas stoves, can change over time and it is important that national surveys continue to ask questions about gas stoves, ventilation, and behaviors related to their use in surveys that also collect information about children’s respiratory health. Additionally, while the type of stoves and heating used in households are often considered by health care providers who are evaluating indoor air quality risk factors in pediatric patients, additional questions relating to the presence of an exhaust fan may provide an opportunity for preventive intervention and improved outcomes.

## Electronic supplementary material

Additional file 1: Figure S1: Description of the population selection criteria used to restrict to children aged 2-16 years of age who live in homes with gas stoves and have complete data for the covariates included in the multivariate regression models. (DOCX 35 KB)

## Abbreviations

CI: Confidence interval
FEV_1_: Forced expiratory volume in 1 second
FVC: Forced vital capacity
NHANES: National Health and Nutrition Examination Survey
NOx: Nitrogen oxides
OR: Odds ratio
P: p-value
PAH: Polycyclic aromatic hydrocarbons
Pct: Percentile
SES: Socioeconomic status.
