# Supplementary material for: A cross-sectional study of the association between ventilation of gas stoves and chronic respiratory illness in U.S. children enrolled in NHANESIII
Source: Environ Health. 2014 Sep 2;13:71. doi: 10.1186/1476-069X-13-71 (PMC4175218; doi:10.1186/1476-069X-13-71)
Supplement: Supplementary file 1 — Additional file 1: Figure S1: Description of the population selection criteria used to restrict to children aged 2-16 years of age who live in homes with gas stoves and have complete data for the covariates included in the multivariate regression models. (DOCX 35 KB) [file 12940_2014_780_MOESM1_ESM.docx]

**Additional file 1: Figure S1.** Description of the population selection criteria used to restrict to children aged 2-16 years of age who live in homes with gas stoves and have complete data for the covariates included in the multivariate regression models.

Asthma
*n=7,378*

Wheeze
*n=7,380*

Bronchitis

*N=7,378*

*Missing BMI (n=1,529)*

*Missing income (n=75)*

*Missing parental asthma Hx (n=12)*

*Missing pets in home (n=17)*

*Missing BMI (n=1,529)*

*Missing income (n=75)*

*Missing parental asthma Hx (n=12)*

*Missing pets in home (n=17)*

*Missing ETS (n=5)*

*Missing income (n=106)*

*Missing parental asthma Hx (n=12)*

*Missing ETS (n=5)*

Asthma sample in
adjusted models
*n=5,745*

Wheeze sample in
adjusted models
*n=5,744*

Bronchitis sample in
adjusted models
*n=7,255*

NHANES III Youth File (all children aged 0-16)

*n = 13,944*

*Excluded children <2 yr old (n=1,339)*

*Excluded participants who lived in homes without gas stoves (n=5,178)*

*Excluded participants who did not provide information on gas stove use (n=35)*

*Excluded participants who did not provide information on ventilation (n=12)*

Children aged 2-16 yrs living in homes with gas stoves with data on gas stove use and ventilation
*n= 7,380*

*Missing information on asthma (n=2)*

*Missing information on bronchitis (n=2)*
